# Birds of a Feather Laugh Together: An Investigation of Humour Style Similarity in Married Couples

**DOI:** 10.5964/ejop.v12i3.1115

**Published:** 2016-08-19

**Authors:** Christian Martin Hahn, Lorne John Campbell

**Affiliations:** a Department of Psychology, The University of Western Ontario, London, Canada; Department of Psychology, University of Western Ontario, London, Canada

**Keywords:** relational humour, self-esteem, marriage

## Abstract

The present research investigated the degree of similarity in humour styles between spouses as assessed with the Humour Styles Questionnaire (HSQ). Furthermore, self-esteem was investigated as a potential moderator of partner humour style similarity. A sample of 116 heterosexual, married couples independently completed questionnaires assessing self-reported humour styles across the 32 item HSQ, as well as global self-esteem. Results indicated that there is significant positive association between the humour styles of married partners. This association was moderated by individual self-esteem. Specifically, participants with high self-esteem were found to have greater humour style similarity with their partners. Similarity was also greater for positive compared to negative types of humour. Implications for the use of dyadic data in investigating the roles of humour within couples are discussed.

Humour is widely regarded as a desirable quality that most people look for in a romantic partner (Feingold, 1992; Goodwin & Tang, 1991; McGee & Shevlin, 2009; Sprecher & Regan, 2002). Indeed, when asked, individuals report that displaying humour is the most effective method of attracting a mate (Buss, 1988). Beyond initial attraction, sharing humourous experiences and laughing together also increases feelings of closeness (Fraley & Aron, 2004). More specifically, romantic partners who share similarities in what they find to be humourous tend to see their partners in a more favourable light and have greater optimism for the future of their relationship (Barelds & Barelds-Dijkstra, 2010; Murstein & Brust, 1985). This may be the case because similarities in humour may be representative of similarities in personal values, needs, and preferred methods of communication, factors that have been demonstrated as important in mate selection (e.g., Abel, 2002; Driver & Gottman, 2004; Kelly, 2002).

The majority of research on humour and mate selection has focused on the relationship outcomes of humour use (e.g., Barelds & Barelds-Dijkstra, 2010; Campbell, Martin, & Ward, 2008; Campbell & Moroz, 2014). There are however, broad themes in regard to what people do and do not find attractive. Among these is the well documented finding that the majority of individuals seek out mates who they find to be similar to themselves (Barelds & Barelds-Dijkstra, 2007; Byrne 1971; Byrne & Nelson, 1965; Klohnen & Luo, 2003). This similarity-attraction hypothesis would suggest that individuals may be drawn to romantic partners whose humour style is similar to their own. However, there are other factors that may influence the degree to which individuals value similarity in a partner. Key among these is self-esteem. Those with higher self-esteem are more likely to seek a partner who is similar to themselves than are those with lower self-esteem (Brown & Brown, 2015; Jones, Pelham, Carvallo, & Mirenberg, 2004). The present study assessed the self-reported humour styles of romantic partners and predicted that there would be similarity in humour style between partners. Furthermore, we assessed self-esteem and predicted that self-esteem would moderate the degree to which partners are similar in humour style.

## Humour and Mate Selection

Humour plays an active role in both interpersonal attraction and mate selection (Buss, 1988; Fraley & Aron, 2004; McGee & Shevlin, 2009; Sprecher & Regan, 2002). This importance of humour in mate selection may be due to the perception that displays of humour are demonstrations of other qualities such as agreeableness (Cann & Calhoun, 2001) and sociability (Wanzer, Booth-Butterfield, & Booth-Butterfield, 1996), which are important factors in relationship satisfaction, conflict resolution, and commitment. However, individuals vary in how they display humour themselves and in how they prefer others to display it (Barelds & Barelds-Dijkstra, 2010; Martin et al., 2003). Though humour may be ubiquitous in its appeal, it follows that individual differences in types of humour typically used may be associated with preferences for the type of humour that they find attractive in a mate.

The multidimensional construct of humour has been deconstructed and categorised in a number of ways, leading some researchers to ask whether or not there are certain aspects of humour that are more attractive than others? The answer to this question is “yes”. Using Martin et al.’s (2003) multi-dimensional humour styles questionnaire, prior research has identified that the two positive humour styles (i.e., affiliative and self-enhancing) are associated with increased positive mood, increased romantic interest, and higher ratings of suitability for a long-term relationship (DiDonato, Bedminster, & Machel, 2013; Howland & Simpson, 2014). The two negative humour styles (i.e., aggressive and self-defeating) are associated with feelings of rejection, feelings of sadness, and lower ratings of suitability for a long-term relationship (DiDonato et al., 2013; Kuiper, Kazarian, Sine, & Bassil, 2010; Winterheld, Simpson, & Oriña, 2013).

Although these humour styles may be broadly viewed as being more or less attractive relative to one another, individual differences persist. The investigation of these differences has yielded further information regarding why individuals may be attracted to certain displays of humour but not to others. Among these is similarity in humour. Prior research has identified similarity between potential romantic partners in the type of jokes told and response to external humourous stimuli to be predictive of attraction (Barelds & Barelds-Dijkstra, 2010; Murstein & Brust, 1985). In the context of humour, how similar we see others to ourselves seems to play a role in how we evaluate them as potential mates.

The tendency for individuals to be attracted to those who are perceived to be similar reflects the similarity-attraction hypothesis (Byrne 1971; Byrne & Nelson, 1965). This hypothesis has received considerable empirical support (e.g., Byrne 1971; Byrne & Clore, 1970). Given that, overall, individuals are more attracted to similar others, it is unsurprising that individuals ultimately tend to choose mates who are similar as well (Epstein & Guttman, 1984). This phenomenon, termed “assortative mating”, has been investigated rigorously in relationship sciences. Although the existing humour literature has established some role of similarity in attraction, this body of work has not specifically investigated whether or not this phenomenon translates into the establishment of romantic relationships between those who share a similar sense of humour. The theoretical rationale for such an association is clear: humour is widely viewed as an attractive quality, individuals place a great deal of value on humour in potential romantic partners, individuals are more drawn to a sense of humour similar to their own, and broadly speaking, individuals tend to form relationships with those who are seen as being similar to themselves.

## Self-Esteem in Assortative Mating

A substantial body of research has investigated the possible mechanisms of action behind assortative mating. One such explanation is that individuals feel more at ease with those who share our ideals, preferences, and interests (Cooper & Sheldon, 2002). Having shared or similar interests and perspectives is closely related to both how attractive and how trustworthy potential romantic partners are perceived (Singh et al., 2015; Ziegler & Golbeck, 2007). Another explanation is that assortative mating is partially guided by self-enhancement bias (i.e., the tendency to view oneself in an overly positive manner; Kwan et al., 2004). Brown and Brown (2015) put forth the notion that because most individuals have a favourable opinion of themselves, these individuals are drawn to romantic partners who mirror their own attributes (Jones et al., 2004) but that those who have an unfavourable view of self would be less drawn to similar romantic partners. Using a small university student sample, Brown and Brown (2015) asked participants to describe themselves, their ideal partner, and to complete a self-esteem questionnaire. The authors found that individuals with higher self-esteem were more likely to describe their ideal romantic partner in terms similar to those used to describe themselves than were their low self-esteem counterparts. This finding is important as it goes a step further than the similarity-attraction hypothesis and demonstrates the influence of self-enhancement bias in mate selection.

The idea that self-enhancement plays a role in mate selection follows the rationale that individuals view their romantic partners as extensions of the self and therefore those who do not like themselves place less value on similarity between a partner and the self. Therefore, it appears that the role of similarity in attraction may not be as simple as once thought but rather is influenced by perceptions of the self. A logical extension of Brown and Brown (2015) is to examine these phenomena in a higher-powered sample of partnered, as opposed to single, participants. There are many possible avenues through which to pursue this investigation. Humour lends itself nicely to this question given the existing literatures establishing the link between mate preferences and attraction, and humour style similarity (e.g., Barelds & Barelds-Dijkstra, 2010; Byrne, 1971). Establishing whether or not similarity in humour styles pervades beyond mate preference and attraction represents an important first step. A follow-up to this is to examine the potential mechanisms of action through which this may occur. Self-enhancement linked to self-esteem may provide one such explanation given the established link between these constructs and the value of similarity in an ideal romantic partner.

## The Current Study

The current study sought to test hypotheses regarding the similarity between humour styles of romantic partners and the role that individual differences (i.e., self-esteem) play in moderating this degree of similarity. Prior research has established strong support for the notion that not only is humour an important characteristic in a potential romantic partner but furthermore that this attraction is increased when a potential partner has a sense of humour that is seen as similar to one’s own. Drawing on established theory, contemporary research in this field has identified self-esteem to play an important role in how similar individuals want their ideal partner to be to him- or her- self. The existing body of research has focused on humour in attraction and the role of similarity therein but has not addressed whether or not these findings carry forward to established relationships. Therefore, the present study provides an important extension by assessing the similarity of humour styles between romantic partners using the Humour Styles Questionnaire (HSQ; Martin et al., 2003), a widely used and well validated scale that assesses the degree to which individuals subscribe to each of four humour styles. We hypothesize that overall, romantic partners will share significant similarity across the humour styles endorsed. We then assessed the influence of individual self-esteem on the association between partner humour styles. We hypothesize that those with higher self-esteem will share greater similarity in humour styles with their romantic partners than will their lower self-esteem counterparts. Findings of this study may provide a fuller understanding of humour in romantic partners that goes beyond existing empirical research investigating humour in romantic attraction.

## Method

### Participants

One hundred and sixteen married heterosexual couples participated in the present study and were compensated with $50.00 per individual ($100.00/couple). Couples were recruited using advertisements in local community newspapers in the city of London, Canada, an urban city with a population of approximately 366,000 that is located southwest of Toronto. The average length of dating prior to marriage was 35.19 months (*SD* = 27.79) and the average length of marriage at time of participation was 120.23 months (*SD* = 127.12). The average total length of relationship was 153.16 months (*SD* = 124.58). The average age of male participants was 38.56 years (*SD* = 11.22) and for female participants was 36.7 years (*SD* = 10.71). The majority of couples (60%) had children (*M* = 1.31 per couple). This data set has been used previously in study 2 of Lackenbauer and Campbell (2012), Campbell, Overall, Rubin, and Lackenbauer (2013), and Campbell and Moroz (2014), but those investigations did not test the hypotheses addressed in the present study.

### Procedure

Data used in the present study were collected as part of a larger study. Only the procedure relevant to the present study will be described herein. Couples were invited to complete their participation at an in-person laboratory session at the University of Western Ontario. At this session, members of the couple were separated and asked to independently provide their informed consent to participate and to complete a set of paper-and-pencil questionnaires. These questionnaires included measures of humour use and perceptions of the self.

### Materials

#### Humour Styles Questionnaire (HSQ)

The HSQ (Martin et al., 2003) assesses self-reported styles of humour use. This 32-item scale measures the degree to which individuals display each of four styles of humour use: affiliative (e.g., I enjoy making people laugh), self-enhancing (e.g., if I am feeling depressed, I can usually cheer myself up with humour), aggressive (e.g., if someone makes a mistake, I will tease them about it), and self-defeating (e.g., I let people laugh at me or make fun at my expense more than I should). There are 8 items for each humour style. This measure uses a seven-point likert scale response mechanism (1 = *totally disagree;* 7 = *totally agree*) and generates scores for each humour style by averaging responses across each 8-item set. Martin et al. (2003) report acceptable reliability (α > .77) and construct validity (based on correlations with peer-report humour style; *r*s = .22 - .33). The internal consistency in the present sample was acceptable for affiliative (α = .85 and .85 for men and women, respectively), self-enhancing (α = .83 and .87 for men and women, respectively), aggressive (α = .78 and .67 for men and women, respectively), and self-defeating (α = .83 and .82 for men and women, respectively) humour styles.

#### Rosenberg Self-Esteem Scale (RSE)

The RSE scale (Rosenberg, 1965) assesses self-reported evaluation of and attitude toward the self. This 10-item questionnaire (e.g., *I take a positive attitude toward myself*) utilizes a four-point likert scale response mechanism (0 = *Strongly disagree,* 3 = *Strongly agree*). Robins, Hendin, and Trzesniewski (2001) report acceptable reliability (α = .88) and construct validity (based on correlation with the Single Item Self-Esteem Scale [Robins, Hendin, & Trzesniewski, 2001]; *r*s = .72 - .76). Participant self-esteem scores are obtained by totalling responses on all ten items (maximum score = 30) with higher scores indicating higher self-esteem. The internal consistency in the present sample was acceptable for this measure (α = .88 and .89 for men and women, respectively).

## Results

### Data Analytic Strategy

Models of interest were investigated using multilevel modeling (MLM; Kenny, Kashy, & Bolger, 1998; Raudenbush & Bryk, 2002), in accordance with the recommendations of Kenny, Kashy, and Cook (2006; see also Campbell & Kashy, 2002). Data in the present study have a nested structure. Specifically, participant self-reported ratings of humour styles across the 32 items of the HSQ (Level 1) are nested within couple (Level 2). We ran two models: Model 1, the test of our primary hypothesis, estimated the within-couple association between participants’ humour styles within couples across the 32 items on the HSQ. This model was run twice, once using male participant HSQ scores as the criterion, and once using female participant HSQ scores as the criterion. Model 2, the test of our secondary hypothesis, estimated the degree to which the association between partner humour styles within-couples is influenced by self-esteem. Model two was run twice, once using male partners’ self-esteem scores, and once using females partners’ self-esteem scores. All predictor variables were grand mean centered for analyses.

### Descriptive Statistics

Descriptive statistics, including response ranges, means, and standard deviations, and t test results for male and female responses to each of the 32 HSQ items are reported in Table 1. For several of these items, significant differences were found between genders. The majority of these differences were found in items corresponding to the negative humour styles (i.e., aggressive and self-defeating). In their initial standardization sample Martin et al. (2003) found significant differences between genders for all subscales. However, it was noted by these authors that strong significance was found only for the negative humour styles and that for positive humour styles, the threshold of significance was barely crossed and this was attributed to the large sample size rather than a true sex difference.

**Table 1:** Descriptive Statistics for the 32-Items of the Humour Styles Questionnaire and t-test Results for differences Between Genders

		Male Partner			Female Partner			
Humour Style	Item	*R*	*M*	*SD*	*R*	*M*	*SD*	*t*
Affiliative	1	1 – 7	6.16	1.26	1 – 7	6.22	1.26	0.32
	5	1 – 7	5.17	1.49	1 – 7	5.03	1.50	0.72
	9	1 – 7	4.96	1.82	1 – 7	5.41	1.44	2.23*
	13	2 – 7	6.27	1.03	2 – 7	6.23	1.14	0.30
	17	2 – 7	5.69	1.58	1 – 7	5.54	1.68	0.73
	21	4 – 7	6.39	0.72	1 – 7	6.23	1.19	1.37
	25	1 – 7	6.07	1.45	2 – 7	6.40	1.07	2.22*
	29	1 – 7	5.29	1.73	1 – 7	4.97	1.76	1.32
Self-enhancing	2	1 – 7	5.05	1.68	1 – 7	5.16	1.65	0.45
	6	1 – 7	5.82	1.41	1 – 7	5.54	1.43	1.52
	10	1 – 7	4.52	1.63	1 – 7	4.53	1.68	0.10
	14	1 – 7	5.45	1.45	1 – 7	5.21	1.58	1.27
	18	1 – 7	4.39	1.63	1 – 7	4.34	1.78	0.10
	22	1 – 7	3.63	1.68	1 – 7	3.64	1.77	0.00
	26	1 – 7	5.24	1.55	1 – 7	5.11	1.60	0.65
	30	1 – 7	5.47	1.46	1 – 7	5.16	1.67	1.73
Aggressive	3	1 – 7	3.65	1.88	1 – 7	3.28	1.78	1.63
	7	1 – 7	3.39	1.68	1 – 7	3.28	1.68	0.53
	11	1 – 7	3.17	1.85	1 – 7	2.40	1.46	3.95***
	15	1 – 7	2.93	1.76	1 – 7	2.42	1.83	2.22*
	19	1 – 7	4.01	1.92	1 – 7	3.20	1.87	3.38**
	23	1 – 7	4.22	1.70	1 – 7	4.06	1.78	0.78
	27	1 – 7	2.59	1.79	1 – 7	2.10	1.59	2.41*
	31	1 – 7	3.99	1.95	1 – 7	3.38	1.91	2.54*
Self-defeating	4	1 – 7	3.34	1.73	1 – 7	2.95	1.78	1.81
	8	1 – 7	3.13	1.88	1 – 7	2.46	1.87	2.59*
	12	1 – 7	4.09	1.84	1 – 7	3.49	1.86	2.95**
	16	1 – 7	3.91	1.90	1 – 7	3.10	1.79	3.49***
	20	1 – 7	2.59	1.58	1 – 7	2.05	1.44	2.81**
	24	1 – 7	2.91	1.63	1 – 7	2.86	1.84	0.22
	28	1 – 7	3.83	1.82	1 – 7	3.49	1.97	1.30
	32	1 – 7	4.09	1.69	1 – 7	3.28	1.77	3.68***

*Note.* *R* = range of scores from 1-7. Degrees of freedom for t-tests = 114.

*p < .05. **p < .01. ***p < .001.

### Hypothesis 1: Partners Will Endorse Similar Humour Styles

Table 2 displays the results for analysis of Model 1. This analysis revealed a significant association across the 32 HSQ items within couples. This is to say that when looking at the responses of married partners to each of the 32 items of the HSQ, the responses endorsed by partner one positively predicted the responses endorsed by his or her partner. The differences in coefficients obtained with male versus female HSQ entered as the criterion are attributable to random error. The results of both analyses are significant at *p* < .001.

**Table 2:** Multilevel Models Testing the Effects of One Partners’ HSQ Responses Predicting the Other Partners’ HSQ Responses (Model 1)

	Model 1									
	Female Partners’ HSQ as Criterion					Male Partners’ HSQ as Criterion				
Variable	*b*	*SE*	*t*	95% CI	*r*	*b*	*SE*	*t*	95% CI	*r*
Partner HSQ Responses	.48	.03	19.20***	.43, .52	.31	.41	.02	20.73***	.37, .44	.33

*Note*. Unstandardized regression coefficients are reported. Approximate effect sizes were computed using the formula *r* = √(*t^2^*/(*t^2^* + *df*)) (see Rosenthal & Rosnow, 2007). Degrees of freedom were 3575.

***p < .001.

### Hypothesis 2: Self-Esteem Will Have a Moderating Effect

**Figure 1:**
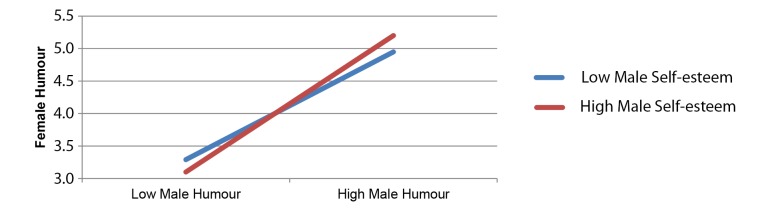
Moderation effects of male self-esteem in the association between male humour (predictor) and female humour (criterion).

**Figure 2:**
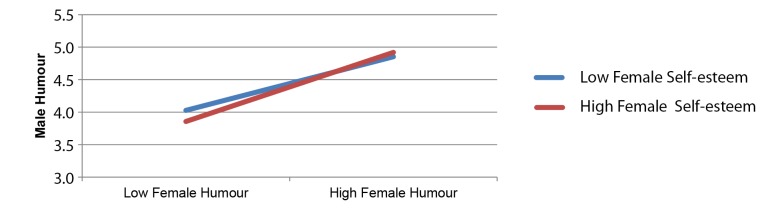
Moderation effects of female self-esteem in the association between female humour (predictor) and male humour (criterion).

Model 2 was run in order to determine whether or not individual differences in self-esteem moderated the association between the humour styles of male and female partners. To test our hypothesis, Model 2 was run twice. First, with male partner humour style as the criterion, and female humour style and female self-esteem as predictors. Second, with female partner humour style as the criterion, and male humour style and male self-esteem as predictors. As illustrated in Table 3, in both analyses the association between humour styles was moderated by self-esteem. Figures 1 and 2 illustrate the associations between partner humour styles for high (+1 standard deviation above the mean) and low (-1 standard deviation below the mean) self-esteem. For both females (Figure 1) and males (Figure 2) with high self-esteem, the association between self and partner humour styles was stronger than for their low self-esteem counterparts.

**Table 3:** Multilevel Models Testing the Effects of One Partners’ HSQ and Self-Esteem Predicting the Other Partners’ HSQ Responses (Model 2)

	Model 2									
	Female Humour as Criterion					Male Humour as Criterion				
Predictor	*b*	*SE*	*t*	95% CI	*r*	*b*	*SE*	*t*	95% CI	*r*
Partner HSQ Responses	.47	.02	19.38***	.42, .52	.31	.40	.02	20.73***	.36, .44	.33
Partner Self-esteem	.01	.07	.21	-.12, .14	.02	-.02	.07	-.38	-.15, .11	.04
Partner HSQ Responses x Partner Self-esteem	.05	.02	2.17*	.01, .09	.03	.05	.02	2.52*	.01, .08	.04

*Note*. Unstandardized regression coefficients are reported. Approximate effect sizes were computed using the formula *r* = √(*t^2^*/(*t^2^* + *df*)) (see Rosenthal & Rosnow, 2007). Degrees of freedom ranged between 113 (self-esteem) and 3574 (humour style) for models estimated with all couples.

*p < .05. **p < .01. ***p < .001.

### Exploratory Analyses

Although not a primary interest at the outset of the study, additional analyses were conducted to investigate potential differences attributable to humour type (i.e., positive and negative) in the similarity between spouses’ humour styles. Specifically, we investigated whether or not the findings above differed for positive (i.e., affiliative and self-enhancing humour styles) versus negative (i.e., aggressive and self-defeating) humour styles. For this analysis, humour type was effect coded such that the 16 positive humour items were each coded as “1” whereas the 16 negative humour items were each coded as “-1”. The models presented in Table 2 were re-run, this time adding the effect code of humour type, as well as the interaction between this effect code and partner HSQ responses (for both male scores and female scores as the predictor). Both male partner humour (*b* = .23, *SE* = .02, *t*(3573) = 9.95, *p* < .001) and humour type (*b* = .94, *SE* = .03, *t*(3573) = 30.91, *p* < .001) were significantly associated with female partner humour. A significant interaction was found between predictor variables in this model (*b* = .04, *SE* = .02, *t*(3575) = 2.74, *p* = .006), suggesting that similarity between male and female HSQ scores was stronger for the positive (*b* = .27) versus negative humour styles (*b* = .19). This analysis was repeated with male partner humour style as the criterion, and female partner humour style and humour type as the predictors. Again, both predictors were significantly associated with the criterion (female partner humour style: *b* = .22, *SE* = .02, *t*(3573) = 10.74, *p* < .001; humour type: *b* = .67, *SE* = .03, *t*(3573) = 21.08, *p* < .001). The interaction between these predictors was significant (*b* = .06, *SE* = .02, *t*(3573) = 4.01, *p* < .001), again suggesting that similarity between female and male HSQ scores was stronger for the positive (*b* = .28) versus negative humour styles (*b* = .16). A final exploratory analysis was run to investigate a potential three-way interaction between partner humour style, humour type, and self-esteem. The results of this analysis were not significant.

## Discussion

The present study was conducted in order to answer two questions: are married partners similar in self-reported humour style? If so, is this similarity moderated by the self-esteem of either partner? In answering the first question, our results are the first to show a strong positive correspondence within couples between the humour styles endorsed by heterosexual married partners, confirming our primary hypothesis. That is, the profiles of self-reported humour across the 32 items of the HSQ, a well-validated hallmark measure of individual humour use, are positively associated between spouses.

The reasons for this association may be complex and the results of our exploratory analysis may help to provide a clearer explanation. These analyses revealed that the overall association between partners’ humour styles was greater in the presence of higher endorsement of positive (i.e., affiliative, self-enhancing) as opposed to negative (i.e., aggressive, self-defeating) humour styles. One possible explanation for this difference is that it is a reflection of the social desirability of different humour styles. Cann and Matson (2014) found that, among dating partners, positive humour styles are viewed as more socially desirable and perceived to be associated with more prosocial behaviours than are their negative counterparts. The greater desirability of, and importance placed on positive humour styles may result in increased likelihood of romantic pairing between individuals with strong endorsement of positive humour styles. This increased likelihood may be the result of an increase in the number of romantic prospects due to the social desirability of positive humour.

An alternate explanation for this finding may be that among established couples, such as those sampled for the present study, the use of positive versus negative humour styles has had relationship enhancing effects. This would imply that couples who express higher endorsement of negative humour styles are less likely to maintain long-term romantic relationships due to the perceptions of expressions of negative humour styles being socially undesirable. Indeed prior research has identified a link between the use of positive humour and relationship satisfaction among dating couples (Caird & Martin, 2014). As evidenced by the significance of similarity between partners endorsing negative humour styles, within dyad humour similarity is ubiquitous. However, it is possible that social desirability or relationship enhancing effects of positive humour is a driving force in the heightened magnitude of humour similarity among couples endorsing positive humour styles.

In answering the second question, our results show that the association found between partner humour styles was indeed moderated by the self-esteem of either partner. Specifically, those with higher self-esteem shared greater similarity in humour style with their partners than did their low self-esteem counterparts. It is, however, important to note that the moderating effect of self-esteem did not alter the direction of association between partners’ humour styles. That is to say that the presence of low self-esteem does not produce an inverse association between partner humour styles (i.e., significantly dissimilar humour styles) but rather lessens the magnitude of the positive association identified above. Prior research has indicated that self-esteem influences the degree to which individuals seek and form romantic relationships with partners viewed as similar to the self (Brown & Brown, 2015). Specifically, that individuals with high self-esteem endorse descriptions of an ideal other that are similar to descriptions of themselves. The present research extends these findings by investigating the role of self-esteem in similarity of established couples, rather than in descriptions of hypothetical partners. This shows evidence that self-enhancement biases pervade beyond who individuals would like to be romantically involved with and into whom individuals go on to form lasting romantic relationships with.

This is the first research to demonstrate a high degree of similarity in the humour styles of married partners. The further exploration of the influence of individual differences (i.e., self-esteem) provides initial evidence of the presence of psychological processes not previously observed in the context of humour (i.e., self-enhancement bias). The results of this study provide a foundation for future works relating to humour in the context of established romantic relationships.

### Strengths and Implications

The use of dyadic data is a major strength of the present study. This allows us to draw objective conclusions regarding the similarity of humour styles among married partners that would not be possible with individual data. For example, having own partner rate his or her own humour style and the humour style of his or her partner may only reflect a perception of similarity that may be motivated by a desire for feelings of relational cohesion. A key implication of this research is that individuals appear to form committed romantic relationships with partners who display a similar humour style. That is, the importance placed on humour similarity seems to extend beyond attraction, as noted in previous literature (e.g., Barelds & Barelds-Dijkstra, 2010). Additionally, this study is among the few extant studies to examine humour in the context of established romantic relationships. This opens up the door for future research investigating the specifics functions served by humour similarity within the dyad.

### Future Directions

The present study has established a clear association of within dyad humour style similarity. Future research should expand beyond the cross-sectional approach used the present study and investigate humour style similarity over time. Studies employing a longitudinal design may identify the dynamic nature of humour styles within couples that is precluded by the nature of the data in the present study. This would identify potential differences in similarity at various stages of the relationship as well as explore a possible trend toward similarity over time.

Further areas for future exploration may involve investigation of the functions that humour and humour similarity may serve within the context of romantic relationships. The present study identifies a strong degree of humour style similarity between married partners but does not explore the possible effects that similarity or dissimilarity may have on relationship outcomes. An intriguing question raised by these findings is “what does this mean for the relationship?”. In answering this question one direction may be to investigate the possible associations between humour style similarity and relationship satisfaction and whether or not these associations are present across all humour styles. Prior research has linked humour use with relationship satisfaction but not necessarily humour similarity (e.g., Aune & Wong, 2002; Caird & Martin, 2014). Furthermore, the present study identifies similarity between married partners in both positive and negative humour, but it is possible that relational benefits that may arise from humour style similarity (e.g., relationship satisfaction) are exclusive to- or are stronger for- partners sharing a positive humour style than for those who share negative humour styles.

### Limitations

The cross-sectional design of the present study prevents us from differentiating between possible directions of causality. For example, it may be the case that individuals are selecting partners because of their similarity in humour or it could be that over time, partners grow increasingly similar in humour style. Longitudinal data that assesses humour style prior to-, at the outset of-, and during a romantic relationship would allow for analysis of changes in individual humour styles over time and changes in the relatedness of humour within the couple. Additionally, by relying on self-reports of humour we may be capturing the styles of humour that participants wish to present or that they believe they display, but not the humour styles that they truly use on a regular basis. Future studies using observational methods may provide insight into the humour styles used by partners when interacting with one another.

### Conclusions

The results of the present study provide a meaningful contribution to the existing literature on humour styles and romantic relationships. By focusing on married couples, we are able to extend established findings on humour similarity beyond the field of initial attraction. In addition, we identified that this similarity is influenced by evaluations of the self. The more positively an individual views him or herself, the more likely there is similarity in humour style between that individual and his or her partner. Overall, we see evidence that humour is an important feature in a romantic partner and that its importance is influenced by factors relating to the self.
